# Electrochemiluminescence Systems for the Detection of Biomarkers: Strategical and Technological Advances

**DOI:** 10.3390/bios12090738

**Published:** 2022-09-07

**Authors:** Seung-Min Yoo, Yong-Min Jeon, Seo-Young Heo

**Affiliations:** School of Integrative Engineering, Chung-Ang University, Seoul 06974, Korea

**Keywords:** electrochemiluminescence, biomarker, detection, luminophore, co-reactant, resonance energy transfer, bipolar electrode

## Abstract

Electrochemiluminescence (ECL)-based sensing systems rely on light emissions from luminophores, which are generated by high-energy electron transfer reactions between electrogenerated species on an electrode. ECL systems have been widely used in the detection and monitoring of diverse, disease-related biomarkers due to their high selectivity and fast response times, as well as their spatial and temporal control of luminance, high controllability, and a wide detection range. This review focuses on the recent strategic and technological advances in ECL-based biomarker detection systems. We introduce several sensing systems for medical applications that are classified according to the reactions that drive ECL signal emissions. We also provide recent examples of sensing strategies and technologies based on factors that enhance sensitivity and multiplexing abilities as well as simplify sensing procedures. This review also discusses the potential strategies and technologies for the development of ECL systems with an enhanced detection ability.

## 1. Introduction

Biomolecules are chemical structures—which can vary from small molecules, such as metabolites, to larger molecules, such as proteins and carbohydrates—that are synthesised by living organisms. The presence and concentration of these molecules remain stable under normal biological conditions. However, any changes in the presence or concentration of specific biomolecules can be related to the malfunction of cells and organisms, which is also closely associated with disease. Therefore, accurately detecting and monitoring the concentration of disease-related biomarkers constitutes a useful strategy for the early detection and treatment of various diseases, which is vital for clinical research, forensics, biodefence and pathology. One of the major challenges when quantifying and detecting biomarkers is the accurate detection of specific targets with a high selectivity in complex biological samples. These biological samples can contain salts, proteins, and other small organic molecules, all of which can hinder the detection-related reactions by acting as inhibitors or competitors. Another major challenge is the quantitative detection of biomolecules with a high sensitivity in a wide range of concentrations. For example, the levels of procalcitonin (PCT), which is a biomarker used in order to diagnose septicaemia, are correlated with the severity of sepsis. A concentration of 0.1 ng/mL or less is generally considered the threshold in order to rule out bacteraemia, whereas 1 ng/mL or more is indicative of the disease. The PCT values between 0.1 and 1 ng/mL can serve as indicators of the disease’s progression depending on the aetiology of the sepsis. However, a sepsis diagnosis and the evaluation of its severity is further complicated by the highly variable and non-specific nature of its signs and symptoms [1,2].

The simultaneous detection of multiple biomarkers (i.e., multiplexing) is also a major challenge, particularly in situations where specimens are in short supply and the same assay volume must be used in a single run. Consequently, many studies have attempted to develop biomarker detection systems using a diverse range of optical, electrochemical, electrical, and mechanical methods [3,4,5,6,7,8,9,10]. Among the various sensing approaches developed thus far, electrochemiluminescence (ECL) has attracted an increasing interest since it provides an effective means of detecting trace concentrations of specific molecules in biological samples. ECL is an analytical technique that combines electrochemical and luminescent methods. Therefore, since it does not require the excitation of a light source, there is no interference from scattered light. This now results in a low background noise and a high sensitivity but also allows for the development of compact and simple devices. In addition to these remarkable advantages, this sensing approach also offers a high selectivity and fast response, as well as the spatial and temporal controls of luminance, an excellent controllability, and a wide detection range [11,12,13]. Several ECL diagnostic kits and systems have since been commercialised by companies such as Radiometer, Roche Diagnostics, Meso Scale Diagnostics, and Biometro (Table 1). Commercialised products consist of either a type of automated analyser or a well plate/kit-based system. The automated analysers, despite their relatively large sizes and high costs, enable the high-throughput detection of biomarkers from a variety of sample types, including serum, cell supernatant, and whole blood, without any pre-treatment of the clinical samples. Well plate/kit-based systems, in contrast, can detect a limited number of biomarkers and/or require the preparation of the sample but they do have a high portability.

Several high-quality reviews have provided detailed summaries of the principles and characteristics of the ECL systems [14,15,16,17]. However, this study focuses on the most recent developments in ECL systems in clinical settings. We introduce several sensing systems that are classified according to the reactions that drive the ECL signal emissions. These include systems based on the chemical reaction between the luminophore and co-reactant, systems that involve the co-reaction accelerator-involved reactions, systems that incorporate resonance energy transfer (RET) reactions, and systems that incorporate signal amplification methods. The different techniques and sensing strategies for the development of the ECL-based biomarker detection systems are discussed in terms of the main determining factors, namely (1) sensitivity, (2) multiplexing ability, and (3) simplicity. We also describe the representative applications of these strategies and technologies and summarise the related parameters such as the linear range and detection limit, among others. Reviewing the applications and recent advancements in ECL detection systems could provide academic and clinical researchers with key insights into the current state of the ECL technology for disease diagnostics. Additionally, we will discuss novel approaches that can improve these ECL systems in order to facilitate their widespread adoption in clinical settings.

## 2. ECL Systems for the Detection of Biomarkers

ECL is a technique used in biomarker detection systems in order to monitor and quantitatively detect the number of analytes by translating a biochemical interaction into an ECL signal. ECL-based detection systems are mainly composed of electrodes and receptors, as well as luminophores which are generally conjugated to a receptor for a specific analyte. The basic sensing principle of this system is that when a potential is applied to an electrode, an electron transfer reaction occurs between the electrochemically excited states of the electrode and the luminophore attached to the receptor, which subsequently emits ECL signals either at or near the electrode. Importantly, this system does not require any external light sources or excitation energy, thus allowing for the biomarker detection without the need for complex and expensive instruments. Furthermore, the minimal photobleaching and low optical background noise achieved with this method results in a high sensitivity with low signal-to-noise ratios.

**Figure 1:**
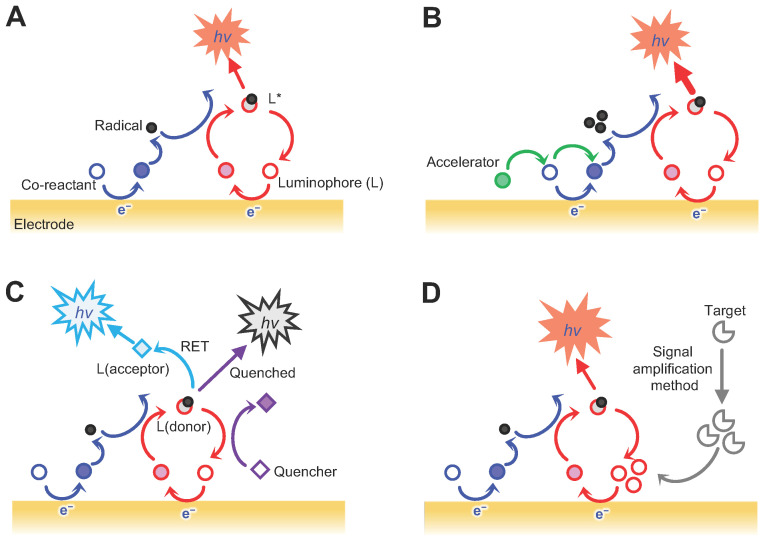
Schematic illustration of EC-sensing systems classified according to the reactions that drive the ECL signal emission. (**A**) Luminophore and co-reactant-involved reaction-based system; (**B**) co-reaction accelerator-involved reaction-mediated system; (**C**) Resonance energy transfer (RET) reactions-incorporated system; and a (**D**) signal amplification method-incorporated system.

There are several sensing systems for medical applications, which are classified according to the reactions that drive the ECL signal emission. These include systems based on the chemical reactions of the luminophores and co-reactants, systems that involve the co-reaction accelerator-involved reactions, systems that incorporate resonance energy transfer (RET) reactions, and systems that incorporate an enzyme reaction-based signal amplification (Figure 1).

The first system is based on the chemical reaction between the luminophore and the co-reactant and is generally used in order to detect diverse biomarkers (Figure 1A). The ECL signals are generated via two different mechanism types depending on the sources of the radicals, which undergo a biomolecular recombination and emit ECL signals. Electrochemical reactions that produce radical species from a single luminophore represent the first route for the generation of an ECL signal, a process referred to as the annihilation mechanism. This mechanism involves the electron transfer between oxidised and reduced species, which are subsequently electrogenerated on the electrode surface through a wide range of potential sweeps. The radical cations and anions produced from the oxidised or reduced species undergo an electron transfer process in order to generate an excited state before relaxing to the ground state and emitting light in the process. Most luminophores generally have a low luminous efficiency meaning that a co-reactant is often used in order to enhance the luminophore’s ECL signal by facilitating the electrochemical reaction between the luminophore and a suitable co-reactant—a second route for the generation of an ECL signal also known as the co-reactant mechanism. The use of a co-reactant is also effective in systems where radical species that are electrogenerated from luminophores have unstable properties and a short lifespan. The co-reactant oxidises or reduces at the surface of the electrode, generating intermediate radicals, which then react with the reduced or oxidised form of the luminophore attached to the receptor. When the analyte is present in the sample, the luminophore attached to the receptor emits photons via electron transfer between the co-reactant and luminophore, thereby generating a visual readout. The co-reactant mechanism proceeds via a sweeping potential in one direction only, using a co-reactant. When selecting the ECL luminophore and co-reactant, it is important to consider both their compatibility with the target analyte and the presence of any cross-interference. Additionally, when selecting external co-reactants, it is essential that their complexability, stability, and reproducibility are also considered, as these factors may affect the analyte detection environment and could potentially leak from the sensor’s interface, particularly when the co-reactant is immobilised on the surface of the sensor [18,19,20].

The second system is a combined reaction involving a co-reaction accelerator. Co-reaction accelerators can be added to the reaction mixture and are involved in the ECL-generating reaction, in which they facilitate the ECL reaction rate of the co-reactant in order to generate a large number of active intermediates (Figure 1B). These effects occur even at low co-reactant concentrations, thereby greatly improving the ECL efficiency [21,22]. Co-reaction accelerators contain metal nanoflowers such as Pt and Ag [23,24], nanoparticles (NPs) such as Au and Fe_3_O_4_ [25,26], graphene nanosheets [27], SnS_2_ nanoplates [28], Pt-Ag alloys [23], metal-organic frameworks (MOFs) [29] and semicarbazide [30]. Moreover, the dual or multiple co-reaction accelerators can be applied in synergy [22,31]. Using accelerators can enhance the sensing capacity of the ECL systems that rely on luminophores with a low luminescent intensity, such as SnO_2_ quantum dots (QDs) [32] because their wide band gap requires a high level of energy in order to achieve the transition of valence electrons from the valence band to the conduction band.

The third system is a combined form that involves the RET reaction (Figure 1C). Unlike a basic ECL signal readout, which uses only one luminophore, the signal can be emitted via the interaction between two different emitters by incorporating a RET [22,33,34]. In such ECL-REL systems, the ECL intensities can vary widely due to the overlapping of the ECL emission spectrum of the donor and the absorption spectrum of the acceptor. Depending on which RET donor-acceptor pair is selected, this approach can resolve some of the major problems of the luminophores, such as the broad spectral emissions, a high compatibility with a wide range of electrode materials or co-reactants, and a low excitation potential. The energy-overlapped RET pairs affect the energy transfer efficiency, which consequently determines the sensitivity of the system. The collision frequency between the RET pair in solution also affects the energy transfer efficiency. The decision of where to conjugate the acceptor and the donor can affect the energy transfer efficiency due to a steric hindrance of the biomolecules in a given sample. Because of this, the position and distance of the acceptor and donor should be optimised in order to increase the collision frequency and minimise energy loss [33]. The introduction of a quencher will endow the ECL-RET system with the ability to modulate the ECL intensity. This strategy is based on the signal change derived from the distance between a RET pair and the quencher, which can be directly added to the reaction mixture [33,34]. Ferrocene and dopamine are representative ECL quenchers used as additives that are functionalised with the receptor. When the sample contains target molecules, the quencher-attached receptor binds to the target, thereby decreasing the distance between the REL pair and the quencher, thus shifting the signal readout to either an ‘on’ or ‘off’ state [33,34]. However, introducing a quencher can lead to more complex operations, higher costs, and a lower labelling efficiency. Quenchers can also be generated in situ through an enzymatic reaction [35]. Such a co-reactant-free system can simplify the detection process of the ECL system.

The fourth system is a combined form that involves an enzyme reaction-based signal amplification method (Figure 1D). Several such methods, such as the hybridisation chain reaction (HCR), enzyme-aided DNA walker signal amplification, 3D nanomachine-based target-recycling reaction, and the catalytic hairpin assembly (CHA), have previously been used in order to detect diverse biomarkers [22,36,37,38,39]. These methods generally rely on the initiation of the binding events between target analytes and probe DNAs, leading to a target-recycling reaction triggered by either enzyme-catalytic reactions or the competitive hybridisation with other ssDNAs with a high affinity toward captured DNAs. During this process, the number of DNA fragments acting as the 2nd target increases, resulting in an increase in the ECL signal. In the case of the enzyme-involved method, the reaction exhibits a high specificity, but the assay time depends on the catalytic ability of the enzymes [40]. The components for the amplification method can be added to the electrodes consecutively, which can complicate the assay process and prolong the assay time. Regarding the hybridisation-based method—and something that should be assessed for all sequence-based methods—the binding efficiency of the nucleic acids or their cross-reactivity should also be considered as it can result in false positive or negative ECL signal readouts. The advantages and disadvantages of the aforementioned systems are summarised in Table 2.

## 3. Current Strategies and Technologies of the ECL Systems in the Detection of Biomolecules

This section highlights the strategies and technologies for overcoming challenges and improving the sensing performance of the ECL biomarker detection systems. The approaches are divided into three main challenges: (1) sensitive detection, (2) multiple detections, and (3) simple detection (see Table 3). The representative applications of these strategies and technologies, including those described in this paper and others that have been developed and employed for the detection of biomarkers, are described in Table 4. The related parameters (linear range, LoD, related diseases, etc.) are also summarised in Table 4.

### 3.1. Strategies and Technologies for the Sensitive Detection of Biomarkers

**Figure 2:**
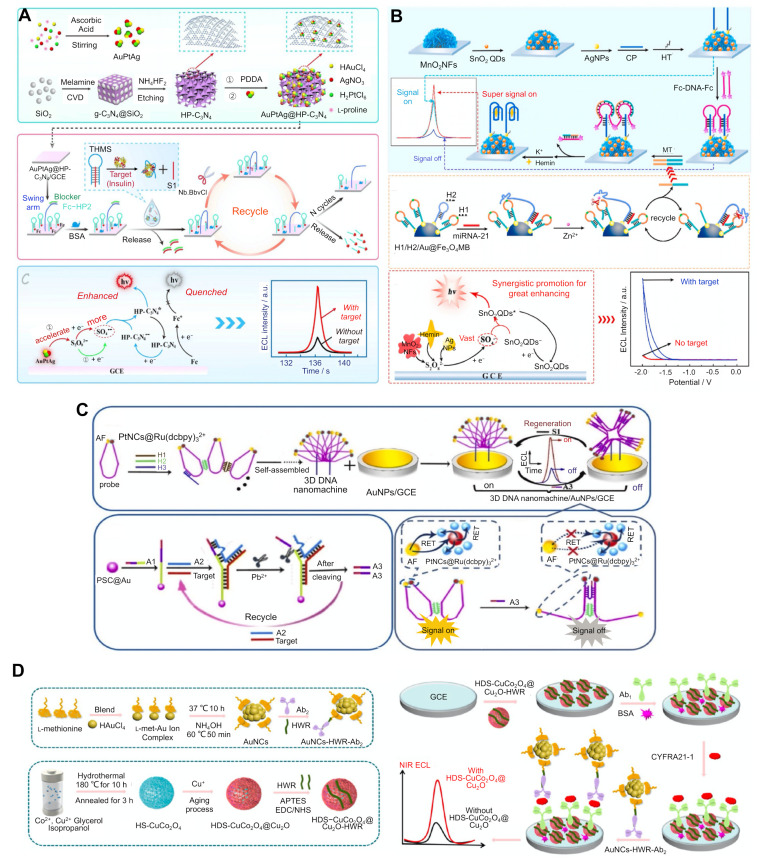
An ECL-based sensing strategy and technology for the sensitive detection of biomarkers. (**A**) Insulin detection system combined with a co-reaction accelerator-involved redox reaction (reprinted with permission from [22], © 2019, Elsevier). (**B**) miRNA-21 detection system combined with a multiple co-reaction accelerator reaction and a 3D DNA walker-mediated signal amplification method (reprinted with permission from [31], © 2020, Elsevier). (**C**) miRNA-141 detection system combined with multiple ECL-RET reactions and a Pb^2+^-dependent DNAzyme-assisted target-recycling amplification (reprinted with permission from [38], © 2020, Elsevier). (**D**) Alpha-fetoprotein antigen (AFP) detection system using CuNCs as a near-infrared (NIR)-ECL luminophore and K_2_S_2_O_8_ as a co-reactant (reprinted with permission from [41], © 2022, American Chemical Society). CVD, chemical vapour deposition; HP, hollow porous; GCE, glassy carbon electrode; BSA, bovine serum albumin; THMS, triple-helix molecular switch; NF, nanoflower; QD, quantum dot; MB, magnetic bead; GCE, glassy carbon electrode; EDC, 1-ethyl-3-(3-dimethylaminopropyl) carbodiimide hydrochloride; NHS, N-hydroxysuccinimide; HWR, Fc-specific heptapeptide with HWRGWVC sequence; HS, hollow sphere.

Several strategies exist for enhancing the limit of detection (LoD) of the sensing systems that use additional components involved in redox reactions and/or are combined with signal amplification methods. One strategy involves the use of a co-reaction accelerator. Co-reaction accelerators promote the ECL reaction rate of the co-reactant in order to generate a large number of active co-reactant intermediates. For example, graphitic carbon nitride (g-C_3_N_4_) has a good biocompatibility, is non-toxic, and is highly stable, making it a promising ECL emitter for biomolecule detection in the medical field. However, its low ECL efficiency limits its practical use. Therefore, instead of bulk g-C_3_N_4_, hollow porous (HP) C_3_N_4_ was manufactured in order to endow it with a high mass transport and systemic reactivity, where protons and electrons can move between the inward or outward pores (Figure 2A) [22]. HP C_3_N_4_ can be further embedded with trimetallic AuPtAg, which acts as a co-reaction accelerator that promotes the ECL reaction rate of the luminophore and co-reactant in order to amplify the signal. AuPtAg@HP-C_3_N_4_ was coated with a glassy carbon electrode (GCE) surface in order to form a sensing substrate. To further improve the sensitivity, we incorporated the Nb.BbvCl-aided DNA walker signal amplification method. For detecting insulin, a AuPtAg@HP-C_3_N_40_ coated GCE substrate was functionalised with two different DNAs: (1) a triple-helix molecular switch (THMS) labelled with ferrocene (Fc) as an ECL emission quencher; and (2) DNA walkers containing a swim arm and a blocker. The triplex-forming molecular beacon (TMS) was composed of both insulin-specific aptamer sequences and stem-forming sequences (S1). If insulin is absent in the sample, Fc prevents the ECL emission from AuPtAg@HP-C_3_N_4_, resulting in an off-state signal. However, if insulin is present in the sample, the aptamer in the TMS specifically binds with insulin and undergoes a conformational change, releasing S1 from the TMS. This released S1 with the sequence complementary to the blocker in the DNA walker forms a duplex with the blocker, which is then removed during washing. The blocker-dissociated swimming arm binds to the ferrocene-labelled strand. Upon exposure to Nb.BbvCl, which generates a nick at a specific sequence in only one strand of DNA on a duplex before digesting it, the Fc-labelled strand is removed, leaving only the swimming arm. This remaining swimming arm binds to another Fc-labelled strand, resulting in multiple cycling reactions of hybridisation and digestion, and eventually switching to the signal-on state. The co-use of AuPtAg@HP-C_3_N_4_ and Nb.BbvCl-mediated DNA walker signal amplification reactions could improve the sensitivity of the system since insulin concentrations as low as 17 fg/mL could be detected with a 0.05 pg/mL–100 ng/mL linear range.

Compared with the use of a single co-reaction accelerator, using multiple co-reaction accelerators can dramatically increase the ECL reaction rate synergistically. To achieve this effect, three types of co-reaction accelerators were simultaneously used in order to facilitate the reduction of the co-reactant (Figure 2B) [31]. For example, all three components (MnO_2_ nanoflowers (NFs), AgNPs, and the hemin/G-quadruplex) can reduce the peroxydisulfate (S_2_O_8_^2−^) co-reactant. With these components, a miRNA detection ECL system could be developed. The sensing substrate was fabricated by functionalising the GCE with nanocomposites consisting of MnO_2_ nanoflowers (NFs; 1st co-reaction accelerator), AgNPs (2nd co-reaction accelerator), and SnO_2_ QDs (a luminophore). Captured DNAs (CPs) were attached to the AgNPs, and the TMS was formed by binding with the DNAs labelled with Fc at both ends. Fc acts as a quencher of the ECL signal from SnO_2_ QDs, which switches the system to an off state. If miRNA exists in the sample, it interacts with the H2 DNA strand attached to the Fe_3_O_4_ magnetic beads (MBs), which are covered with miRNA- and H1 DNA strand-binding sequences.

Binding between the miRNA and H1 renders a hairpin structure of the H1 opening and leads to the sequential binding with H2, which generates the recognition site for the Zn^2+^ ions. Once the Zn^2+^ ions were added, H1 was digested into short fragments, after which it acted as a destabiliser of the TMS on the sensing substrate, resulting in the release of Fc-labelled DNAs. The remaining CPs from the TMS formed a G-quadruplex structure with the added hemin molecules, thus facilitating the ECL emission as a 3rd co-reaction accelerator and switching to the signal-on state. This system exhibited a detection limit of 2.5 aM miRNA, with a linear range of 10 aM to 100 pM.

A quenching strategy was adopted in an ECL-RET system to sensitively detect various biomarkers such as the *p53* gene and retinol-binding protein [42,43,44]. In this ECL system, a lanthanide MOF and S_2_O_8_^2−^ were used as the luminophore and co-reactant, respectively. Crystal violet (CV), which was used as an acceptor of photons and electrons emitted from excited LaMOF, was oxidised, which quenched the ECL emissions, thereby providing the first quenching effect. CV was functionalised with dsDNA because of its high affinity toward dsDNA, which provided the second quenching effect. In order to detect the *p53* gene, CPs were attached to LaMOF with a carboxyl group via the amide coupling reaction. The absence of the *p53* gene kept the LaMOF bound to ssDNA and prevented the reaction when CV was added, resulting in ECL emission. When the sample contained *p53*, the ssDNAs were hybridised with CPs, forming dsDNA, which bound with the CV, decreasing the ECL signal. This system could detect *p53* levels as low as 0.33 fg, with a linear range of 1 pg to 100 nM showing that using a quencher can modulate the ECL intensity as well as sensitively detect biomarkers.

The combination of various signal amplification methods is another strategy that can enhance the sensitivity of the system [33,38]. For example, a sensitive miRNA detection system was developed by combining the ECL-RET and target-recycling reactions (Figure 2C) [38]. In this system, the nanocomposites are formed using Pt nanocrystals as the energy donor and Ru(dcbpy)_3_^2+^ nanocomposites as the energy acceptor, thereby shortening the electron transfer path, reducing energy loss and improving the RET efficiency. Alexa fluor (AF) acts as another donor for transferring energy to PtNC@Ru(dcbpy)_3_^2+^ nanocomposites, generating multiple energy transfer effects. These donor-acceptor pairs are attached to the probe DNA at each end and form a loop structure, thus shortening the distance between AF and PtNC@Ru(dcbpy)_3_^2+^. ssDNA-functionalised probe DNAs are linked via three different ssDNA, H1, H2, and H3, forming a 3-dimensional (3D) DNA nanomachine. When the target is absent from the sample, this 3D nanomachine keeps the ECL signal in the ‘on’ state by maintaining its conformation. If the target is present in the sample, the target bound to A1 ssDNA will attach to PSC@Au and sequentially interact with another A2 ssDNA, forming a Pb^2+^-dependent DNAzyme. Upon adding Pb^2+^, a partial region of the A1-A2 duplex is digested, and the target is released from the structure, binding to another A1 ssDNA, triggering a target-recycling reaction. A3, the part of the ssDNA of A1-A2 duplex cleaved by Pb^2+^, could hybridise with the 3D DNA nanomachine, thus lengthening the distance between AF and PtNC@Ru(dcbpy)_3_^2+^, causing a decrease in the ECL signal and switching the system to an ‘off’ state. This system could detect miRNA concentrations as low as 3 aM by combining multiple ECL-RET reactions and target-recycling reactions. This combination of signal amplification strategies exhibits a high specificity but has additional material and processing costs, the possibility of variations in assay times depending on the catalytic ability of the enzymes and nanozymes, and potential cross-reactivity.

The use of ECL emitters with aggregation-induced and crystallisation-induced emission activity can enhance the sensitivity of ECL systems [45,60]. These emitters include tetraphenyl alkene (TPA) and tetraphenylethylene (TPE) nanocrystals [46]. The ordered structure limits the free rotation of benzene rings in order to suppress the nonradiative transition and facilitates the electron transfer in order to enhance the radiative transition, which enhances the ECL reaction. A previous study employed this strategy in order to detect dopamine [46]. In this system, the TPA nanocrystals and triethylamine were used as the luminophore and co-reactant, respectively. During the electrochemical reaction, the dopamine could be oxidised to benzoquinone, which quenched the ECL signal of the TPA nanocrystals, thus decreasing the signal readout. The ECL signal decreased with higher dopamine levels, achieving an LoD of 3.1 nM, with a linear range of 5 nM to 10 μM.

Efforts have also been made to develop near-infrared (NIR) ECL luminophores with a high versatility and stability, a near zero background signal, and a high controllability [59]. As NIR emissions have a lower background interference, cause less photochemical damage, and have a deeper tissue penetration, NIR-ECL systems have become powerful analytical tools in biomedical and diagnostic fields. The Ru (II) complex [61,62], QDs [63,64], metal NCs [41,47,48,49,65,66], nanocrystals [46,50], and perylene diimide [67] have all been used as NIR-ECL emitters. Tetraphenylenthylene (TPE) NC was synthesised and applied to the aggregation-induced ECL emission method [46]. For example, a ternary metal chalcogenide such as AgInS_2_/ZnS was used as a NIR luminophore in order to develop a NIR-ECL system [47], and the passivation of AgInS_2_ with ZnS with a wide band gap resulted in a high quantum yield [68,69]. Furthermore, in order to detect carbohydrate antigen 125 (CA125, which is a cancer biomarker), GCE was treated with CA125-specific antibodies [47]. Upon exposure to the target, CA125 was captured with specific antibodies and sequentially bound to AgInS_2_/ZnS-labelled antibodies in a sandwich-type ELISA, resulting in an ECL emission. Another example reported the use of AuNC with hollow double-shell (HDS) CuCo_2_O_4_@Cu_2_O heterostructures as a co-reaction accelerator in order to detect the cancer marker CYFRA21−1 (Figure 2D) [41]. In order to detect the biomarkers, GCE was functionalised with HDS-CuCo_2_O_4_@Cu_2_O, which was fused with heptapeptides (HWRGWVC; HWR) capable of binding specifically with the Fc region of the antibodies for a site-specific antibody immobilisation on the substrate. Ab-HDS-CuCo_2_O_4_@Cu_2_O-HWR conjugates on GCE captured target molecules by binding with AuNCs-attached antibodies in a sandwich-type ELISA, showing an LoD of 0.67 fg/mL by observing the ECL emissions at the NIR region. Copper NCs were used as NIR-ECL emitters and were applied in order to detect alpha-fetoprotein [48], which could itself be detected by using AuNCs as a NIR-ECL emitter [49]. The conjugation of NP and NC with capping ligands provided a high stability, biocompatibility, and sensitivity. Conjugating AuNCs with methionine as a stabiliser was used in order to detect alpha-fetoprotein [49]. Moreover, L-glutathione-tagged NCs were used in order to detect a prostate-specific antigen (PSA) [50]. The wide range detection by using both NIR emitters and visible emitters could hold great promise in the development of multivariate analysis.

### 3.2. Strategies and Technologies for Multiple Biomarker Detections

The simultaneous detection of multiple biomarkers and the analysis of samples in a single run and/or a single volume can improve the diagnostic efficiency and reduce the diagnostic costs compared with singleplex assays [60,70,71]. This is particularly important in cases where the samples are either limited or available only in small quantities.

The spatially resolved array-based ECL systems constitute a representative method for multiplexing assays, in which analyte-capturing receptors are immobilised at defined positions on a single substrate and share the same assay volume in a run. This technology has now been commercialised as it is in widespread use. However, this approach often requires additional procedures and expensive materials for the fabrication of microscopically integrated spot arrays and well-designed conductive multiwall plates [51]. Unlike the use of defined positions by dividing the area of a substrate, the use of multiple luminophores is based on a spot-free assay format for multiplexing and its process is equivalent to the workflow of a singleplex assay. The readout of this system relies on the ECL signals from multiple luminophores with distinguishable ECL emission wavelengths in a single assay. In this case, the potential- or wavelength-resolved analyses are generally performed for the multiplex detection instead of intensity-based analyses in which ECL emissions from each luminophore cannot be spatially resolved, resulting in a poor selectivity. A recent study developed a multicolour ECL system using three different luminophores in order to detect three biomarkers: carcinoembryonic antigen (CEA), alpha-fetoprotein (AFP), and beta-human chorionic gonadotropin (β-HCG) [51]. The three different luminophores—Ir(ppy)_3_, Ru(bpy)_2_(dvbpy)^2+^, and Ir(dFCF_3_ppy)_2_(dtbbpy)^+^—emitted ECL signals at different wavelengths and exhibited different signal intensities depending on the applied potentials, even at the same emission wavelength. In this system, one target-specific antibody was attached to several MBs while another target-specific antibody was bound to luminophores. The target biomarker was captured by two antibodies via a sandwich-type ELISA and the captured targets were separated using magnets. Upon exposure to acetonitrile, multiple luminophores were released from the antibody-target complexes and interacted with tri-n-propylamine (TPrA) as a co-reactant, thus emitting an ECL signal. This approach enables us to distinguish multiple biomarkers by analysing multiple ECL readout modes, intensity, spectra, and imaging analyses.

**Figure 3:**
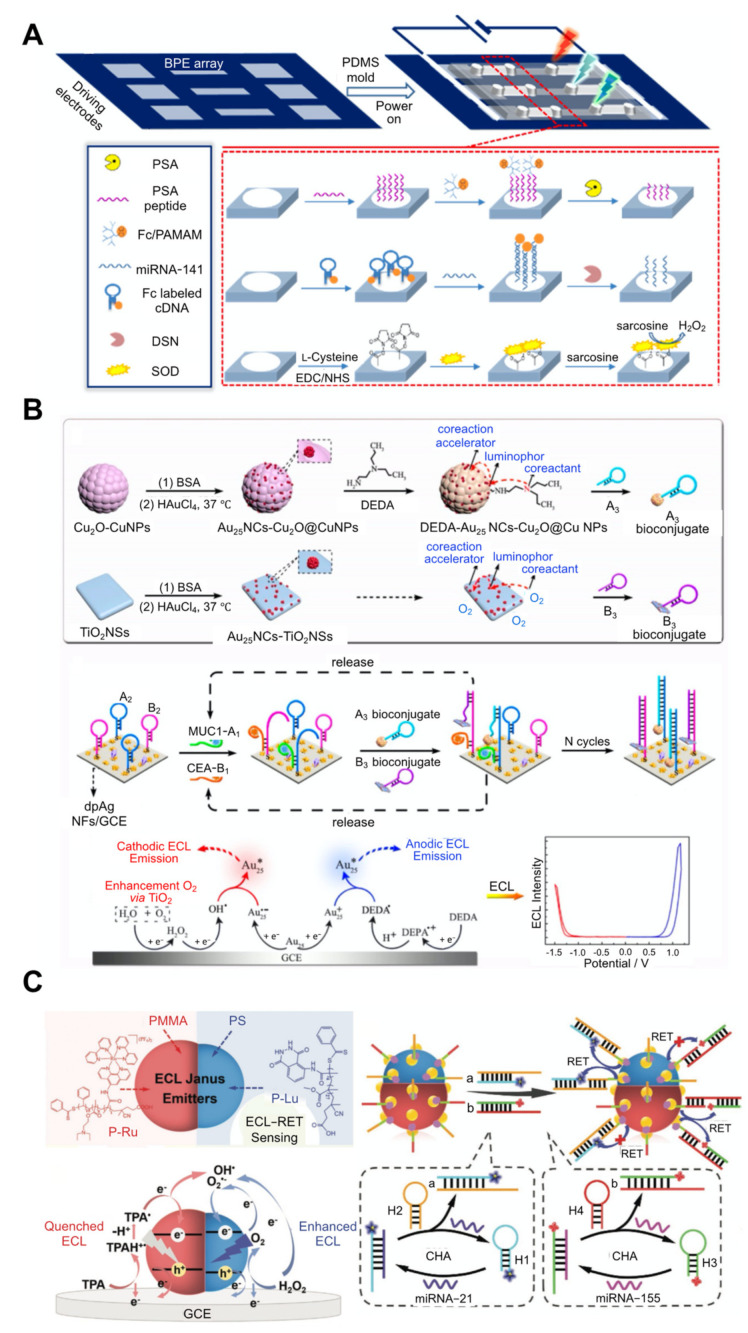
An ECL-based sensing strategy and technology for the detection of multiple biomarkers. (**A**) Prostate-specific antigen (PSA) detection using multiple ECL emitters on a multiple bipolar electrode (BPE) array (reprinted with permission from [53], © 2018, American Chemical Society). (**B**) Carcinoembryonic antigen (CEA) and mucin 1 (MUC1) detection using a bipolar ECL emitter on a single electrode (reprinted with permission from [37], © 2019, American Chemical Society). (**C**) miRNA-21 and miRNA-155 detection using the Janus NP emitter as a luminophore (reprinted with permission from [39], © 2022, Wiley-VCH GmbH). DSN, duplex-specific nuclease; SOD, sarcosine oxidase; BSA, bovine serum albumin; DEDA, N, N-diethylethylenediamine; NS, nanosheet; NP, nanoparticle; NC, nanocluster; PMMA, poly (methacrylic acid methyl ester); GCE, glassy carbon electrode; CHA, catalytic hairpin assembly; RET, resonance energy transfer.

Alternatively, multiplexing detection can also be achieved by employing one ECL emitter on a bipolar electrode (BPE), in which the ECL emitter can be used either as a multicolour or bipolar luminophore. BPEs, and closed BPEs in particular, possess two physically separated zones in one substrate, namely a reporting and sensing zone, and can thus minimise the interference of the environmental factors that could disturb the signal readout. Many studies have attempted to develop a multicolour ECL emitter showing various colours by controlling the applied potential, which enables the development of a current-resolved device based on the closed BPE structure [53,58,72,73]. Among these multicolour ECL emitters, Ru(bpy)_3_^2+^ and bis(3,5-difluoro-2-(2-pyridyl)phenyl-(2-carboxypyridyl) iridium (III) (Ir(df-ppy)_2_(pic)) were used to simultaneously detect multiple biomarkers, including PSA, miRNA-141, and sarcosine (Figure 3A) [53].

A BPE array was created by depositing polydimethylsiloxane (PDMS) moulds on an indium tin oxide (ITO) substrate in the form of a closed system that physically separated the organic solvent from the target molecules. In a BPE used in the detection of PSA, Fc-poly(amidoamine) (PAMAM) labelled PSA-specific peptides were attached to the cathode pole. The PSA in the sample released Fc-PAMAM from the peptides, resulting in a reduction in the current. In the BPE for the detection of miRNA-141, the Fc-labelled DNA hairpins were attached to the cathode. The presence of miRNA led to the specific binding with DNA hairpins, forming open DNA and DNA-miRNA duplexes. The duplexes could be cleaved by an attack of the added duplex-specific nuclease, which released Fc from the duplex and bound to other Fc-labelled DNA hairpins, facilitating the target-recycling reaction, and leading to a decrease in the current flow. In a BPE for the detection of sarcosine, sarcosine oxidase was attached to the cathode. This enzyme oxidised sarcosine in the sample to H_2_O_2_, which was reduced at the cathode, thereby increasing current flow.

The ECL emitters were added to the anodic poles. After applying a potential for the oxidation of Fc to Fc^+^, the increased Fc concentration increased the current flowing to the system and the ECL colour changed from green to dark pink, red, light pink, greyish, and light blue-green by using TPrA as a co-reactant under the control of the current flow. This mixture affected the behaviour of the bidirectional ECL colour change (blue-green to red to light blue-green). This system could detect PSA, miRNA-141, and sarcosine concentrations as low as 4.0 ng/mL, 20 fM, and 1.0 M, respectively. In a similar method, which used a PDMS-based ECL-BPE array chip, multiple prostate cancer biomarkers including PSA, interleukin-6 (IL-6), and a prostate-specific membrane antigen (PSMA) were detected [52]. This system exhibited a LoD for PSA, IL-6, and PSMA which are 0.093 ng/mL, 0.061 pg/mL, and 0.059 ng/mL, respectively.

The bipolar ECL emitters exhibit noninterfering dual-polar ECL emissions under cathodic and anodic scanning. A luminophore can induce a dual-band ECL emission at two wavelengths under chemical reactions employing different co-reactants and co-reaction accelerators. Bipolar ECL emitters were used as luminophores in order to detect multiple biomarkers [32,37,54,74]. For example, Au_25_ nanoclusters (NCs) exhibit cathodic and anodic ECL emissions using different co-reactants and co-reaction accelerators [75]. The Au_25_ NCs were applied to the detection of two biomarkers, a carcinoembryonic antigen (CEA) and mucin 1 (MUC1) (Figure 3B) [37]. In this case, the sensing platform was fabricated on the GCE by electrodepositing with AgNFs and modifying with the stem-looped A2 and B2 ssDNAs, each of which consists of sequences that complement A1 (MUC1-specific aptamer), in addition to A3 for A2, and B1 (CEA-specific aptamer) and B3 for B2. Au_25_NCs were used as the cathodic ECL emitter by integrating them with TiO_2_ nanospheres (NSs) as the co-reaction accelerator using the dissolved O_2_ in a solution as the co-reactant, forming Au_25_NCs-TiO_2_NSs. The combination of Au_25_NCs with Cu_2_O-CuNPs exhibited an anodic ECL emitter, which uses diethylethylenediamine as a co-reactant, forming Au_25_NCs-Cu_2_O@CuNPs. Subsequently, Au_25_NCs-TiO_2_NSs and Au_25_NCs-Cu_2_O@CuNPs were functionalised with A3 and B3 ssDNA with stem-loop structures, respectively. If there were no biomarkers in the sample, the biomarker-specific aptamers kept their conformation and did not bind to A2 and B2 on the electrode. Additionally, A2 and B2 also kept their stem-looped structure and could not bind to the A3-attached Au_25_NCs-Cu_2_O@CuNPs and the B3-attached Au_25_NCs-TiO_2_NSs, which prevented cathodic and anodic ECL emissions. However, if the sample contained biomarkers, their binding stretched the structure of the stem-looped aptamers, which were sequentially bound to A2 and B2 on the electrode, forming a duplex. Upon exposure to the A3-attached Au_25_NCs-Cu_2_O@CuNPs and the B3-attached Au_25_NCs-TiO_2_NSs, they detached A1 and B1 from the duplexes and the released A1 and B1 could bind to another A2 and B2 on the electrode, resulting in a signal amplification via the target-recycling reaction. This system could detect CEA and MUC1 levels as low as 0.43 pg/mL and 5.8 fg/mL, respectively.

Janus NPs were also used as luminophores [39,76] and were applied in the detection of multiple biomarkers [39]. These NPs possess two or more distinct physical and chemical properties in a single unit, such as anisotropic composition, morphology, and surface chemistry [77]. The different properties are spatially separated in one particle as asymmetric heterostructures, which can reduce the interference from signals generated by each property [78,79]. One representative ECL Janus emitter was created by coupling a luminol polymer (P-Lu) loaded polystyrene (PS) compartment and a ruthenium (II) complex (P-Ru) loaded polymethylmethacrylate (PMMA) compartment and was applied in order to simultaneously detect two different miRNAs (miRNA-21 and miRNA-155) in an ECL-RET system (Figure 3C) [39]. The Ru-Lu Janus emitter exhibited blue and red photoluminescence (PL) emissions from the P-Lu and P-Ru on each hemisphere of the Janus NP, respectively. The absorption spectrum of the P-Ru and the emission spectrum of the P-Lu overlapped, resulting in a RET with no signal interference between the two ECL signals because of their spatial separation. For the simultaneous detection of two different miRNAs, the Janus NPs were modified with AuNPs, and different DNAs were captured (D1 and D2). This system was combined with the CHA amplification method in order to amplify the signal readout. If the miRNAs were absent in the sample, two different stem-looped DNAs (FAM-labelled H1 and Cy5-labelled H3) kept their structure and no binding events with capture DNAs occurred. This resulted in a dual-colour readout since no ECL signals were quenched by FAM or Cy5. However, if the sample contained miRNAs, then H1 and H2 bound with these miRNAs, forming duplexes. The added stem-looped DNAs (H2 and H4) released miRNAs from the duplexes, which could bind then with another H1 and H3, resulting in the target-recycling reaction. The multiple generated dye-attached H1-H2 and H3-H4 duplexes bound with D1 and D2, causing quenching by FAM and Cy5, thereby reducing the ECL signals from the Janus NPs. Using this strategy, miRNA 21 and miRNA 155 could be detected at concentrations as low as 8.7 × 10^−15^ M and 1.2 × 10^−15^ M, with a linear range of 10 × 10^−15^ to 10 × 10^−9^ M.

### 3.3. Strategies and Technologies for the Simple Detection of Biomarkers

**Figure 4:**
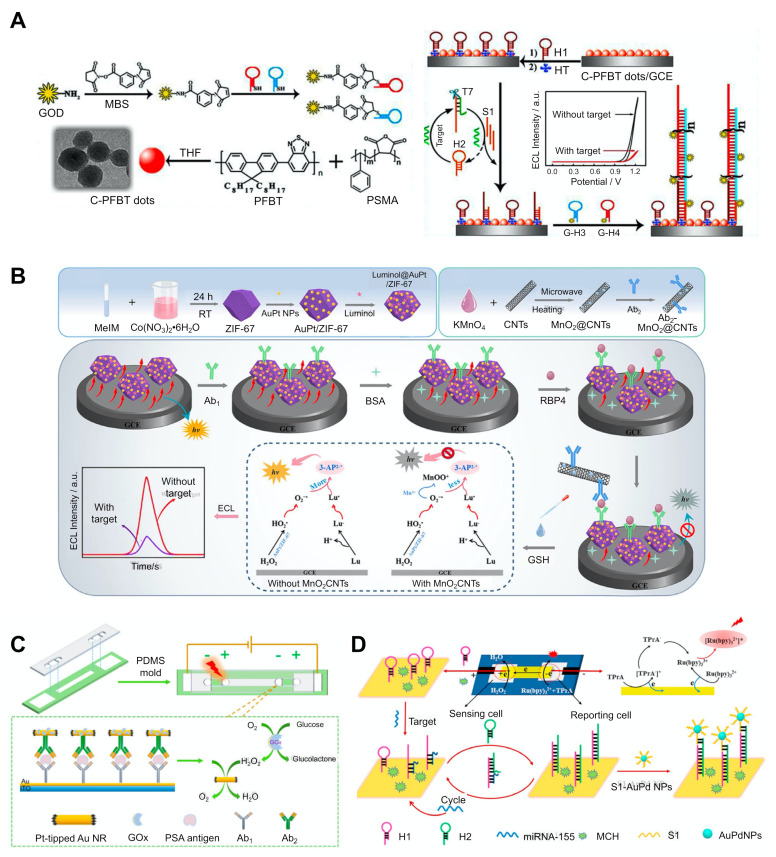
ECL-based sensing strategy and technology for the simple detection of biomarkers. (**A**) miRNA detection using a co-reactant-free system combined with an in situ generated quencher reaction (reprinted with permission from [35], © 2020, Elsevier). (**B**) Retinol-binding protein 4 (RBP4) detection using the nanozyme-catalytic reaction-based system (reprinted with permission from [44], © 2021, Springer Nature). (**C**) Prostate-specific antigen (PSA) detection system using a polydimethylsiloxane (PDMS)-based bipolar electrode (BPE) (reprinted with permission from [56], © 2019, American Chemical Society). (**D**) miRNA-155 detection using a paper-based BPE-ECL system (reprinted with permission from [57], © 2020, American Chemical Society). C-PFBT, carboxyl-functionalised poly[(9,9-dioctylindole-2,7-diyl)-co-(1,4-benzo-{2,1′-3}-thiadiazole)]; PSMA, poly-(styrene-co-maleicanhydride); GCE, glassy carbon electrode; CNT, carbon nanotube; GSH, glutathione; NR, nanorod; GOx, glucose oxidase; MCH, 6-mercapto-1-hexane; NP, nanoparticle.

Biomarker detection can be achieved quickly and easily by implementing an approach known as a component-free system, so-called because it does not require the components involved in a typical ECL emission-generating reaction such as co-reactants, quenchers, and enzymes. This component-free system does not require the addition of external components and thus the process is relatively simple and easy to operate. One approach is to incorporate a quencher-free, co-reactant-free method into the ECL-RET system [35,55]. As discussed above, the use of a quencher enables a variable ECL intensity, which depends on the distance between the RET pair and the quencher in the ECL-RET system. Most systems achieve this quenching effect by adding the quencher to the electrode, although this often leads to methodological complications and increased costs [33,34]. In order to overcome this issue, a system for generating quenchers in situ has been proposed [35]. For example, in situ generated H_2_O_2_ by glucose oxidase acted as a quencher of the light emission of the strong ECL emitter poly[(9,9-dioctylindole-2,7-diyl)-co-(1,4-benzo-{2,1′-3}-thiadiazole)] (PFBT) dots (Figure 4A) [35]. In this system, a PFBT dot-doped GCE substrate was functionalised with hairpin DNA (H1). If the sample contained miRNA, it could bind with another hairpin DNA molecule (H2), resulting in a stretched H2 structure and the production of a DNA:RNA hybrid with a partial duplex. This partial duplex could be cleaved by adding T7 exonuclease, which specifically digests the blunt 5′ termini of the DNA region in the duplexes, releasing miRNA from the duplex and triggering the target-recycling reaction. The remaining DNA part from the T7 exonuclease (S1) could bind to H1 on the GCE substrate, generating the stretched H1. This H1 could then sequentially bind to the glucose oxidase (GOx)-labelled H3 and the GOx-labelled H4 in order to initiate the HCR. Given that Gox catalysed the reaction, H_2_O_2_ could be produced and quenched the ECL emission of the PFBT dots. This system could detect levels as low as 33 aM by implementing enzyme-catalysed quenchers in situ along with a dissolved oxygen-based co-reactant.

The enzyme-free strategies can also be used in order to develop simple systems. One strategy is to use enzyme-mimicking NPs, which are also known as nanozymes [44,56]. The catalytic activity of some NPs is similar to that of enzymes, which triggers redox reactions and energy transfer, which in turn induces ECL emissions from the luminophore. Compared with natural enzymes, using nanozymes can reduce the costs associated with the synthesis and mass production and provides several advantages such as the resistance to harsh environments (e.g., pH and temperature) and facile functionalisation [80,81,82]. Nanozymes include peroxidase, oxidase, catalase, and hydrolase mimics such as Fe_3_O_4_, metallic oxide NPs (Co_3_O_4_ and CeO_2_), and noble metal NPs (AuNPs, Ag/PtNPs, and Au/PdNPs). For example, an AuPt/ZIF-67 NPs-based ECL system was developed in order to detect the retinol-binding protein 4 (RBP4), a type 2 diabetes mellitus-specific biomarker (Figure 4B) [44]. In this system, the AuPt/ZIF-67 NPs system acted as an ECL donor by producing peroxidase activity-mediated H_2_O_2_. It also acted as a support matrix to facilitate the conjugation of antibodies. In particular, its porous structure and incorporation of NPs provided a large surface area, which enhanced the loading of antibodies and the ECL signal. In order to detect miRNA, AuPt/ZIF-67 NPs were first conjugated with a luminophore and sequentially bound with RBP4-specific antibodies. If there was no RBP4 in the sample, luminol@AuPt/ZIF-67-Ab emitted the ECL signals. However, if the sample contained RBP4, luminol@AuPt/ZIF-67-Ab could bind with the target. This complex could then bind with another RBP4-specific antibody, which could attach to carbon nanotubes (CNTs) functionalised with MnO_2_. MnO_2_ provides a quenching effect by reacting with glutathione, generating Mn^2+^ and inhibiting the peroxidase activity of nanozymes [83,84].

The overlapping spectra of luminol emissions and MnO_2_ absorption enables the ECL-RET, which provides an additional quenching effect [85]. The presence of RBP4 resulted in the recruitment of an MnO_2_ quencher and a decreased ECL signal. This system could detect as little as 43 fg of miRNA.

Facile detection can also be achieved by fabricating polydimethylsiloxane (PMDS)-based BPE [36,53,55,58]. The readout relies on different ECL signals from luminophores at both poles during cathodic and anodic scanning. Therefore, despite the use of multiple luminophores, it can prevent the cross-reaction caused by using multiple luminophores in a single-step detection method. This method generally uses a microchannel with two poles on a flat substrate. When an external voltage is applied, the ECL readout is generated through the redox events that occur at both ends of the BPE [56,58].

The sensing performance of this system depends on several factors, such as the rate of the reduction reaction, the driving voltage, and the carrier for loading luminophores on both sides. Using this system, the presence or absence of PSA in several samples could be simultaneously analysed (Figure 4C) [56]. The PSA-specific antibodies were immobilised with 3-aminopropyltriethoxysilane-coated ITO and the samples were injected into a microfluidic channel. If the sample contained PSA, it was captured with the specific antibodies on the ITO substrate, after which they bound to another specific antibody that was labelled with AuNPs. Following the introduction of a silver enhancement solution, silver was deposited on the AuNPs and linked to the two ITO bands, resulting in increased resistance of the BPE. A mixture of Ir(ppy)_3_ and Ru(bpy)_3_^2+^ with TPrA as the co-reactant was added to the anodic reservoir. The cathodic reservoir contained an aqueous solution of a phosphate buffer with NaCl. The sandwich binding at the cathode facilitated the production of the Gox-catalysed H_2_O_2_ production and reduction, balancing with the oxidation of luminophore and co-reactant at the anode, and emitting an ECL signal. This process occurred in the microchannel after the injection of the sample without additional input of any components.

A recent study reported on a paper-based BPE-ECL system that could detect specific biomarkers [36,57]. This BPE-ECL system could be integrated into a portable device that would not require a direct electrical connection or an external power supply. However, the patterning process of the reporting and sensing port on the ITO substrate is complicated and time-consuming and can require expensive instruments such as a photolithography system. However, the easy reduction of H_2_O_2_, which can damage the ITO, also limits its practical use and widespread adoption. The use of paper as a substrate provides several advantages and facilitates the development of simple, low cost, disposable, portable and biocompatible sensors with easy-to-immobilise receptors [86,87,88,89]. Moreover, its porous structure and hydrophilic properties mean it can be used as an ECL sensing platform in order to detect biomarkers such as miRNA-155 (Figure 4D) [57].

The fluid line on the closed BPE was fabricated via conductive carbon ink-jet printing in order to separate the reporting and sensing zones. The hairpin DNA (H1) was attached to the cathode and the miRNA. The binding of the miRNA and H1 initiated the target-recycling reaction and led to the sequential binding between H1 and the added hairpin DNA (H2). Upon adding S1 ssDNA-attached AuPd NPs, AuPd catalysed the H_2_O_2_ reduction and the ECL luminophore emitted the signal at the cathode based on the charge balance at the cathode and anode. The system exhibited an LoD of 0.67 pM, with a linear range of 1 pM to 10 μM. In a similar device, two different miRNAs were multiply detected [36]. In this system, CdTe QDs and Au@g-C_3_N_4_ NSs were used as dual ECL emitters and the carboxylated Fe_3_O_4_ magnetic nanoparticles as carriers and could detect values as low as 5.7 fM of miRNA-155 and 4.2 fM of miRNA-126 by combining with a 3D nanomachine-mediated signal amplification process.

## 4. Conclusions and Perspectives

Over the past few decades, since ECL was initially discovered in 1927, ECL techniques have been rapidly adopted and continuously improved in order to develop biomarker detection systems. This review summarised the recently developed ECL systems that can detect disease-related biomarkers.

Many strategies to improve the performance of ECL systems have already been proposed and many more are being actively developed for the development of highly sensitive, specific, simple, multiplexed, and cost-effective assays, as discussed in this review and summarised in Table 2 and Table 3. Even though tremendous advances have been achieved in recent years, commercially available ECL products are still quite limited in scope (Table 1). The use of paper-based portal devices and the combination of microfluidics mentioned above may facilitate the commercialisation of ECL technology for the detection of biomarkers.

However, there are still challenges that will need to be addressed in the future before ECL use becomes widespread. One such challenge is the development of a luminophore with distinctive characteristics. Specifically, an ideal luminophore would possess a high quantum efficiency, broad spectral emissions, highly stable electrogenerated species, adequate aqueous solubility, low cost, low excitation potential, and a high compatibility with a wide range of electrode materials or co-reactants. Additionally, an ideal luminophore would also prevent electrode fouling, as well as possess a narrow band gap that requires low energy in order to achieve the transition of valence electrons from the valence band to the conduction band. Various luminophores have been developed, from classic emitters such as inorganic metallic/organometallic complexes and organic polyaromatic hydrocarbons to metal oxides, perovskite, upconversion nanoparticles, QDs, and MOFs [90,91]. Moreover, in order to improve the ECL efficiency, luminophores have been designed with confined structures, which provide a high level of mass transport and a long lifespan, in addition to their inherent persistent properties. Another study reported that hollow porous NPs exhibited enhanced ECL efficiency because of their reduced inner filter effect and their minimisation of inactive ECL emitters [22,92]. However, although most commercially available ECL systems currently use limited luminophores such as Ru(bpy)_3_^2+^, the continuous development of ECL emitters with superior characteristics that can be manufactured at a lower cost may facilitate the commercialisation of many more ECL systems.

Another challenge in the establishment of biomarker detection systems is to develop novel co-reactants in order to improve luminophore sensitivity and stability. Biomarkers are commonly detected in an aqueous solution meaning that the components that generate the ECL signal should be highly soluble and stable in water. A high biocompatibility and low toxicity are important factors for any ECL detection system used in medical applications. As mentioned above, Ru(bpy)_3_^2+^ is among the most widely used luminophores and is often used in conjunction with diverse co-reactant amines such as TPrA, S_2_O_8_^2−,^ and H_2_O_2_. These chemicals limit the practical use of the sensors due to their toxicity, volatility, corrosiveness, and instability [92,93,94,95]. Many efforts have also been made, therefore, to identify novel co-reactants in order to overcome these limitations [59] and to improve ECL efficiency. For example, N-hydroxysulfosuccinimide (NHSS) was used as a co-reactant in order to detect proline and Hg^2+^ [59]. NHSS was also effective in detecting biomolecules with amine functional groups such as amino acids compared with various amines such as TPA. Along with the development of novel luminophores, novel co-reactants can also provide an ECL system with superior abilities such as an improved sensitivity and a dynamic detection range.

Developing miniaturised and portable point-of-care ECL systems for widespread use in real-world applications is another problem that must be resolved. In order to achieve this, the ECL imaging capturing apparatus such as PMT and CCD should be substituted for a simpler and more cost-effective system—preferably one that does not need an external power supply. RGB analysis software-embedded smartphones would be an option for capturing the signal and calculating its intensity. Additionally, the integration of customised apps in smartphones can transfer data wirelessly and/or on IoT devices.

Paper- and cloth-based ECL systems have been developed as a way of reducing manufacturing costs [36,57,96]. The screen-printing technique has been widely used to fabricate paper and cloth-based ECL devices. Previous screen-printing methods have several fabrication steps, resulting in a complex, costly, and time-consuming process. Simple and straightforward screen-printing methods will need to be developed in order to allow for the one-step manufacturing of ECL systems. Recently, 3D-printed ECL platforms were introduced for titanium and pyrolytic graphite sheets [17,97,98], the technology of which can allow rapid prototyping and a one-step fabrication process with a high repeatability and reproducibility. Although 3D printing still has its limitations, such as poor resolution and multi-material printing as well as the flexibility in the applications of printing materials with different characteristics, it may eventually facilitate the large-scale production of ECL systems if the technology advances sufficiently.

The development of biomarker-specific receptors will also play an increasingly critical role in the advancement of ECL sensing technologies for biomarker detection in clinical and environmental settings. Most ECL systems, including all currently available commercialised systems, are based on immunoassays that use antibodies. Although immunoassays can provide sensitive and specific results, the labelling and bioconjugations of antibodies are limited and the orientation immobilised on the electrode or its surface should also be considered as it can lower the sensitivity of the system. In this aspect, an aptamer can be an option in order to overcome the limitations. Aptamers are short, synthetic nucleic acids that can selectively bind to various target molecules including small chemicals, proteins, and even whole cells [99,100,101,102,103,104,105]. In addition, they are easy to synthesise and modify with label tags or functional groups either within or at the ends of the strands. They also exhibit a high affinity, high specificity, high thermostability, high acid-base resistance, low immunogenicity and toxicity, and are cheap to manufacture. Aptamers with a high affinity can be discovered through an iterative in vitro process known as the systematic evolution of ligands by exponential enrichment (SELEX). Improving the SELEX method would allow for the widespread use of aptamers as a receptor in the development of highly sensitive and specifically, low cost ECL systems.

Taken together, the advancements described in this review will undoubtedly contribute to the development of more accurate, sensitive, efficient, and cost-effective ECL systems for the detection of disease-related biomarkers in the medical field.

## Figures and Tables

**Table 1:** Commercialised products and assay kits for the biomarker detection.

Company	Product	Representative Biomarkers	Related Diseases	Features	Website
Radiometer	AQT90 FLEX analyser	Troponin, creatine kinase-muscle/brain, myoglobin, N-terminal-pro brain natriuretic peptides, procalcitonin, C-reactive protein, D-dimer, human chorionic gonadotropin, etc.	Sepsis, heart failure, myocardial infarction, venous thromboembolism.	No sample preparation is necessary; Immunoassay; Applications for emergency sample assay; Results in less than 21 min; Automated analyser.	https://www.radiometeramerica.com/ (accessed on 31 August 2022)
Roche Diagnostics	Cobas analyser	High-sensitive troponin T, creatine kinase, myoglobin, parathyroid hormone, human chorionic gonadotropin, etc.	Anaemia, cardiac and tumour markers, critical care, fertility/hormones, infectious diseases	Immunoassay; Applications for emergency sample assay; Throughput of up to 86 tests/h; Automated analyser.	https://diagnostics.roche.com/global/en/products/instruments/cobas-e-411-ins-502.html (accessed on 31 August 2022)
Meso Scale Diagnostics	Meso Sector analyser	SARS-CoV-2, calprotectin, platelet-derived growth factor receptor-β, insulin-like growth factor binding protein 4, angiotensin-converting enzyme 2, Tau, lymphotactin, etc.	Alzheimer’s disease, cardiovascular disease, bone disorders, cardiac biomarkers, cardiac injury, kidney injury, liver injury, muscle injury, cancer	Immunoassay; Use of 96-well or 364-well plates; Throughput of up to 50 plates/h.	https://www.mesoscale.com/ (accessed on 31 August 2022)
Biometro	Lucia system	C-reactive protein.	Inflammation	Immunoassay; Cathodic ECL system; Portable analyser.	http://www.biometro.net/en/ (accessed on 31 August 2022)

**Table 2:** Advantages and disadvantages of the biomarker detection ECL systems.

Sensing System	Advantages	Limitations
Systems based on the chemical reactions of the luminophore and co-reactant	Simple design and operation; Rapid detection; Multiplexing capability using dual or multiple ECL emitters.	Limited sensitivity; Possibility of cross-interference between the luminophore and co-reactant; Possibility of a low stability and reproducibility of the electrode immobilised co-reactant.
Systems that incorporate co-reaction accelerator-involved reactions	High sensitivity by using dual or multiple co-reaction accelerators; Easy selection of luminophore regardless of the luminescent intensity.	Need for an additional synthesis and conjugation process of the co-reaction accelerator.
Systems that incorporate resonance energy transfer reactions	High sensitivity, Extension of the dynamic range of the modulation in the ECL intensity by introducing a quencher.	Potential of varying the energy transfer efficiency depending on the position and distance of the acceptor and donor; Complex operation; Additional costs for materials and processing.
Systems that incorporate an enzyme reaction-based signal amplification	Superior sensitivity.	Complex operation; Additional costs for materials and processing; Potential variations in assay time depending on the catalytic ability of enzymes and nanozymes; Potential cross-reactivity in the hybridisation-based method.

**Table 3:** Challenges and strategies for improving the performance of the biomarker detection ECL systems.

Challenge	Performance Improvement Strategy	References
Sensitivity	Use of a co-reaction accelerator; Use of nanomaterials or nanostructures with hollow porous structures; Combination of RET systems; Combination of signal amplification methods; Use of nanomaterial-aggregation-induced ECL emission methods; Use of NIR-ECL emitters.	[22,32,37,41] [32,41] [39,42,43,44] [22,32,37,39] [45,46] [41,46,47,48,49,50]
Multiplexing capability	Adapting an electrode array with a 3D nanostructure as a substrate; Use of multiple ECL emitters; Use of dual-polar ECL emitters based on different co-reactants and co-reaction accelerators; Use of Janus NP as an ECL emitter.	[51,52] [53] [37] [39]
Simple operation	Implementation of the host-guest inclusion-based bioprobing technique (no need for bioconjugation); Use of a co-reactant-free system; Use of a nanozyme-catalytic reaction-combined enzyme-free system; Use of a PDMS-based BPE system; Use of a paper-based BPE-ECL system; Implementation of microfluidic devices.	[45,54] [35,55] [56,57] [36,52,53,56] [36,57] [36]

**Table 4:** Examples of electrochemiluminescence systems for detecting diverse biomarkers a.

Electrode	Co-Reactant	Luminophore	Receptor	Target	Related Disease	Dynamic Range	LoD	Sample	Features	Reference
GCE	S_2_O_8_^2^^−^	Hollow porous C_3_N_4_	Apt	Insulin	Diabetes	0.05 pg/mL–100 ng/mL	17 fg/mL	Human serum	Use of AuPtAg NP as a single type of co-reaction accelerator. Combination of Nb.BbvCl-aided DNA walker signal amplification methods.	[22]
GCE	S_2_O_8_^2^^−^	SnO_2_ QDs	Capture DNA	miRNA-21	Cancer	10 aM–100 pM	2.9 aM	Cell lysates	Combination with a 3D DNA walker. Use of MnO_2_ NFs, AgNPs and hemin/G-quadruplex as multiple co-reaction accelerators.	[32]
GCE	S_2_O_8_^2^^−^	Lanthanide MOF	Capture DNA	p53 gene	Cancer	1 pM–100 nM	0.33 pM	Human serum	Co-quenching (1) effective ECL-RET quenching between a LaMOFs-CV pair in microchannels; (2) quenching caused by the dsDNA-bridged electron transfer from excited LaMOFs to CV.	[42]
GCE/AuNP	S_2_O_8_^2^^−^	Ru(bpy)_3_^2+^	Ab	NT-proBNP	Heart failure	0.0005 ng/mL–100.0 ng/mL	0.28 pg/mL	Human serum	Use of a PDA-coated Fe_3_O_4_ as a quencher. Use of a AuNP-modified GO-Ru(bpy)_3_^2+^/Ag_2_C_2_O_4_ as a luminophore	[43]
GCE	TEA	TPA nanocrystal	None	Dopamine	Neurological diseases	5 nM–10 μM	3.1 nM	Human serum	Use of a crystallisation-induced enhanced ECL of the TPA nanocrystals.	[45]
GCE	TPrA	Ir(ppy)_3_, Ru(bpy)_2_(dvbpy)^2+^, Ir(dFCF_3_ppy)_2_(dtbbpy)^+^	Ab	CEA, AFP, β-HCG	Cancer	ND	ND	None	Multicolour ECL system using three different luminophores with different emission spectra and a potential resolved ECL generation ability.	[51]
ITO	TPrA	[Ru(bpy)_3_]^2+^ [Ir(ppy)_3_]		PSA	Cancer	1 ng/mL–20 ng/mL	ND	Human serum	Closed BPE system. ECL emission based on modulating the resistance of the BPE.	[58]
ITO	TPrA	Ru(bpy)_3_^2+^, Ir(df-ppy)_2_(pic)	Ab	PSA, miRNA-141, sarcosine	Cancer	1 ng/mL–25 ng/mL for PSA, 10 × 10^−15^ M–10 × 10^−10^ M for miRNA-141, 5 × 10^−7^ M to 5 × 10^−4^ M for sarcosine	4.0 ng/mL PSA, 20 fM miRNA-141, and 1.0 M sarcosine	Human serum	Closed ECL-BPE system. ECL emission based on modulating the resistance of the BPE.	[53]
FTO	TPrA	Ru(bpy)_3_^2+^	Ab	PSA, IL-6, PSMA	Cancer	-	0.093 ng/mL (for PSA), 0.061 pg/mL (for IL-6), 0.059 ng/mL (for PSMA)	Human serum	Closed BPE array microfluidic chip system (3 × 6 array).	[52]
GCE	DEDA (for anodic ECL), dissolved O_2_ (for cathodic ECL)	Au_25_ NC	None	CEA, MUC1	Cancer	ND	0.43 pg/mL (for CEA), 5.8 fg/mL (for MUC1)	None	Use of TiO_2_ NSs and Cu_2_O@CuNPs as cathodic and anodic co-reaction accelerators, respectively. Combination of the target-catalysed hairpin hybridisation as a signal amplification strategy.	[37]
GCE/AuNP		C_60_(ZnTPP)_3_@γ-cyclodextrin	Capture DNA	miRNA	Cancer	1 pM–100 nM	120 fM	Human serum	Use of a host-guest inclusion-based universal probe tag for the ECL signal readout, with no need for a biofunctionalised pre-treatment of the luminophore.	[54]
GCE	TPrA	Ru(bpy)_3_^2+^, luminol	Capture DNA	miRNA-21, miRNA-155	Cancer	10 × 10^−15^ M–10 × 10^−9^ M	8.7 × 10^−15^ M for miRNA-21 and 1.2 × 10^−15^ M for miRNA-155	Cell lysates	Combination of the ECL-RET and CHA reaction. Use of a quenching effect by RET between Janus NPs and dyes (Cy5 and FAM).	[39]
GCE	Dissolved oxygen	C-PFBT dot	None	miRNA	Cancer	ND	33 aM	Cell lysates	No additional input of a co-reactant. Use of in situ generated H_2_O_2_ via GOx catalytic reaction. Combination of a target-recycling reaction and HCR	[35]
ITO	H_2_O_2_	Luminol	Ab	CYFRA 21-1	Cancer	0.0075 ng/mL–50 ng/mL	1.89 pg/mL	Human serum	Use of CaO_2_ possessing a capacity of self-supplying H_2_O_2_ and O_2_ via a hydrolysis reaction of CaO_2_. Use of a BPE-ECL system.	[55]
GCE		Luminol	Ab	RBP4	Type 2 diabetes mellitus	0.0001 ng/mL–100 ng/mL	43 fg/mL	Human serum	Use of luminol@AuPt/ZIF-67 (ECL donor) with peroxidase activity for developing an enzyme-free system. Use of MnO_2_@CNTs and GSH as a quencher (dual quenching system).	[44]
Au/ITO	TPrA	C-Ir (III) for the cathode, (pq)_2_Irbza/TPrA for the anode	PtNR-GOx- Ab	PSA	Cancer	1 pg/mL–10 ng/mL	0.72 pg/mL	None	Use of BPE. Use of Pt-tipped AuNRs for facilitating the reduction of H_2_O_2_.	[56]
Paper	TPrA	Ru(bpy)_3_^2+^	Capture DNA	miRNA-155	Cancer	1 pM–10 μM	0.67 pM	None	Paper-based BPE-ECL system.	[57]
Paper	S_2_O_8_^2^^−^	CdTe QDs, Au@g-C_3_N_4_ NSs	Capture DNA	miRNA-126, miRNA-155	Cancer	1 × 10^−14^ M–1 × 10^−7^ M	5.7 fM (for miRNA-155), 4.2 fM (for miRNA-126)	None	Paper-based dual-channel BPE-ECL system for multiple detections. Combination of Nb.BbvCl-aided DNA walker signal amplification method.	[36]
GCE	TPrA	AgInS_2_/ZnS NC	Ab	Carbohydrate antigen 125	Cancer	5 × 10^−6^ U/mL–5 × 10^−3^ U/mL	1 × 10^−6^ U/mL	Human serum	NIR-ECL system.	[47]
GCE	Tri-isopropanolamine	AuNC	Ab	CYFRA21−1	Cancer	2 fg/mL–50 ng/mL	0.67 fg/mL	Human serum	NIR-ECL system. Use of AuNCs as the luminophore and hollow double-shell-CuCo_2_O_4_@Cu_2_O heterostructures as the co-reaction accelerators	[41]
GCE	K_2_S_2_O_8_	CuNC	Ab	AFP	Cancer	1 ng/mL–400 ng/mL	0.02 ng/mL	Human serum	NIR-ECL system.	[48]
GCE	TEOA	AuNC	Ab	AFP	Cancer	3 fg/mL–0.1 ng/mL	1 fg/mL	Human serum	NIR-ECL system. Use of the methionine-tagged Au NCs to achieve NIR-ECL.	[49]
GCE	TEOA	Ag−Ga−In−S NC	Ab	PSA	Cancer	0.05 pg/mL–1.0 ng/mL	0.01 pg/mL	None	NIR-ECL system. Use of the GSH-tagged NCs.	[50]
GCE	NHSS	Ru(bpy)_3_^2+^	None	L-Proline, Hg^2+^	Cancer	0.5 μM–200 μM (for proline), 0.1 μM–25 μM (for Hg^2+^)	50 nM (for proline), 10 nM (for Hg^2+^)	Serum, urine, lake water	Anodic ECL system.	[59]

^a^ Abbreviations: LoD, limit of detection; GCE, glassy carbon electrode; Ab, antibody, Apt, aptamer; NP, nanoparticle; MOF, metal-organic frame; NT-proBNP, N-terminal pro-B-type natriuretic peptide; TEA, triethylamine; TPA, tetraphenyl alkene; PDA, polydopamine; GO, graphene oxide; NC, nanocrystal; CV, crystal violet; TPrA, tri-n-propylamine; CEA, carcinoembryonic antigen; AFP, alpha-fetoprotein; β-HCG, beta-human chorionic gonadotropin; DEDA, N,N-diethylethylenediamine; NS, nanosheet; MUC1, mucin 1; CYFRA 21-1, squamous cell carcinomas named cytokeratin 19 fragments; miRNA, microRNA; C-PFBT, carboxyl-functionalised poly[(9,9-dioctylindole-2,7-diyl)-co-(1,4-benzo-{2,1′-3}-thiadiazole); GOx, glucose oxidase; FTO, fluorine-doped tin dioxide; iL-6, interleukin-6; PSMA, prostate-specific membrane antigen; HCR, hybridisation chain reaction; RBP4, retinol-binding protein 4; CNT, carbon nanotube; GSH, glutathione; C-Ir(III), cyclometalated iridium(III); PSA, prostate-specific antigen; NR, nanorod; BPE, bipolar electrode; NIR, near-infrared; NC, nanocluster; TEOA, Triethanolamine; NHSS, N-hydroxysulfosuccinimide; ND, not determined.

## Data Availability

Not applicable.
